# Catalytic IgG Antibodies Hydrolyze DNA, Histones, and HMGB1 in Systemic Lupus Erythematosus

**DOI:** 10.3390/ijms26199635

**Published:** 2025-10-02

**Authors:** Mark M. Melamud, Evgeny A. Ermakov, Anna S. Tolmacheva, Irina A. Kostrikina, Alexey E. Sizikov, Georgy A. Nevinsky, Valentina N. Buneva

**Affiliations:** 1 Institute of Chemical Biology and Fundamental Medicine, Siberian Branch of the Russian Academy of Sciences, 630090 Novosibirsk, Russia; marken94@mail.ru (M.M.M.); evgeny_ermakov@mail.ru (E.A.E.); tolmacheva.anna0301@gmail.com (A.S.T.); irina@kostrikina.ru (I.A.K.); alex.sizikov.as@gmail.com (A.E.S.); nevinsky@niboch.nsc.ru (G.A.N.); 2 Department of Natural Sciences, Novosibirsk State University, 630090 Novosibirsk, Russia; 3 Department of Rheumatology, Immunopathology Clinic, Research Institute of Fundamental and Clinical Immunology, Siberian Branch of the Russian Academy of Sciences, 630090 Novosibirsk, Russia

**Keywords:** systemic lupus erythematosus, anti-DNA antibodies, IgG, catalytic antibodies, abzymes, DNase activity, histone, HMGB1, hydrolyzing activity, catalase activity

## Abstract

Antinuclear antibodies, especially anti-DNA antibodies, are known to be a hallmark of systemic lupus erythematosus (SLE) and represent a diverse pool of autoantibodies with different origins, antigenic properties, and physicochemical features. Antibodies with catalytic properties have been found among the antibody repertoire in SLE, but the specific features and clinical associations of such antibodies have not been sufficiently studied. This study showed that chromatographically purified IgG from the serum of SLE patients effectively hydrolyzed DNA and DNA-associated proteins such as histones and high-mobility group box 1 (HMGB1) compared to healthy individuals. Remarkably, the level of hydrolysis of DNA and DNA-associated proteins was closely correlated. At the same time, these antibodies did not hydrolyze the control protein, tumor necrosis factor-α (TNFα), which does not possess DNA-binding properties. IgG DNase activity levels varied significantly, so patients were divided into high- and low-activity subgroups using the DBSCAN algorithm, with the difference between median values being greater than 49 times. The subgroup with high IgG DNase activity was characterized by an increase in anti-DNA antibodies (*p* < 0.04) than the subgroup with low activity, which had a shorter duration of the disease (*p* = 0.03) and was more often characterized by a subacute rather than a non-chronic course of the disease (*p* = 0.048). High catalase-like activity of IgG was also detected in SLE. Thus, the antibody pool in SLE contains not only high-affinity antinuclear autoantibodies but also catalytic antibodies capable of hydrolyzing DNA and DNA-associated proteins. These findings expand our understanding of the heterogeneity of the repertoire of catalytic autoantibodies among SLE patients.

## 1. Introduction

Systemic lupus erythematosus (SLE) is a systemic diffuse autoimmune disease that radically affects the quality of life of patients. The prevalence of SLE is between 3 and 517 cases per 100,000 population, depending on the region [1]. According to a systematic review, approximately 34% of patients with SLE acquire a work disability [2]. The treatment of SLE patients is also associated with a high economic burden [3]. Thus, the social consequences and high economic costs of treatment dictate the need to study the mechanisms of SLE pathogenesis in order to develop new therapeutic drugs and diagnostic strategies.

The immunopathogenesis of SLE is associated with the combined influence of genetic and environmental risk factors contributing to the disruption of immunological tolerance and development of autoimmune response [4]. Impaired clearance of apoptotic cells, excessive netosis, and complement dysfunction are associated with the accumulation of autoantigens in SLE [5,6]. Abnormalities in T-cell subpopulations, regulatory T-cell dysfunction, and altered cytokine regulation contribute to the pathogenesis of SLE [7,8]. However, the central element of SLE pathogenesis is impaired B-cell development and function, leading to the production of autoantibodies that form immune complexes and contribute to tissue damage [9].

One of the pathognomonic signs of SLE is known to be the formation of autoantibodies to DNA (anti-DNA antibodies), which contribute to tissue damage, including the development of lupus nephritis [10,11,12]. Extracellular double-stranded DNA (dsDNA) released in complex with histones and other nuclear proteins is supposed to be one of the antigens stimulating the formation of anti-dsDNA antibodies [13,14]. Anti-DNA antibodies are also capable of internalizing into cells, promoting apoptosis and inflammation [15,16,17,18]. The DNA-anti-dsDNA antibody immune complexes can contribute to netosis activation and release of nuclear components, further stimulating the formation of antinuclear antibodies [12,19].

In addition to DNA and histones, other DNA-associated proteins can be released into the extracellular space during cell death. One such protein is high-mobility group box 1 (HMGB1). HMGB1 is a non-histone protein involved in maintaining the structure of nucleosomes and packaging DNA into chromatin [20]. Extracellular HMGB1 can also act as an alarmin and perform cytokine-like functions promoting inflammation [21]. HMGB1, along with DNA, histones, and their complexes, can act as an antigen, promoting the formation of autoantibodies. There is evidence that anti-HMGB1 antibody levels are elevated in approximately half of SLE patients, especially with lupus nephritis [22,23,24]. Thus, chromatin components are a rich source of a wide range of autoantibodies, but the mechanisms of their generation and pathological effects are not yet fully understood.

A wide range of autoantibodies differing in target antigen, affinity, and properties are generated in SLE [25,26,27]. Antibodies with non-canonical properties, in particular catalytic antibodies, have also been identified among the repertoire of autoantibodies [28,29,30]. Such antibodies bind antigen, although with somewhat lower affinity, and catalyze biochemical reactions, including hydrolysis, oxidation, and others, expanding the functional capabilities of immunoglobulins [31,32,33,34]. The ability to catalyze is determined by the unique structure of the fragment antigen-binding region, formation of structural motifs similar to enzymes (including catalytic triads), coordination of metal ions involved in nucleophilic catalysis, and others [35,36,37]. Catalytic antibodies are found in many diseases, including SLE [28,30,38]. Natural catalytic antibodies hydrolyzing DNA [39,40,41], RNA [42,43], microRNA [44], polysaccharides [45], histones [46], and other proteins [46,47,48] have been shown in several studies to be detectable in SLE. In addition, catalytic antibodies with redox activities [49,50,51], including catalase-like activity [52,53], have been found in healthy individuals and patients with other diseases, indicating a possible contribution to protection against oxidative stress [54]. However, antibodies with redox activity have not been studied in SLE, although oxidative stress is known to occur in this disease [55,56]. Moreover, the heterogeneity of catalytic antibodies and clinical associations in SLE patients are largely unexplored.

This study investigated catalytic antibodies directed against DNA and DNA-associated proteins (histones and HMGB1), as well as IgG with catalase-like activity in SLE patients and healthy subjects. Clinical associations with antibody catalytic activity levels have also been investigated. This study showed that the IgG of SLE patients hydrolyzes DNA and DNA-associated proteins in SLE. The ability of IgG to hydrolyze HMGB1 was identified for the first time. In addition, it was shown for the first time that the IgG of SLE patients exhibited high catalase-like activity. The data also highlighted the heterogeneity of SLE patients in terms of catalytic activity levels.

## 2. Results

### 2.1. Clinical Characteristics of Sample

The sample of this study included 56 SLE patients and 35 healthy subjects (HS) (Table 1). Only women were included, as SLE is more common in women. The age of the participants did not differ significantly in the SLE and HS groups (Table 1). Expectedly, SLE patients had a higher concentration of anti-ssDNA and anti-dsDNA IgG in serum than controls. Median disease duration was 8.5 years. The median SELENA-SLEDAI score was 6. Most patients (96%) were in the active phase of disease. Approximately half of SLE patients (49%) had moderate SLE activity, although there were patients with high (16%), low (2%), and minimal activity (33%). The majority of patients (71%) had a chronic course of SLE.

### 2.2. IgG Isolation from Serum and Confirmation of Purity and Homogeneity of the Obtained Samples

Samples of circulating polyclonal IgGs were purified from serum using a previously developed method of affinity chromatography followed by High-Performance Liquid Chromatography (HPLC) (see Section 4.2). In previous studies, we have shown that this method provides highly purified IgG samples free of any impurities [52,57,58], as also confirmed by mass spectrometry [57]. We have also previously provided evidence that IgG samples have a number of catalytic activities [42,49,52,58]. In this work, we also confirmed the homogeneity and purity of the analyzed IgG samples by gradient (4–18%) denaturing electrophoresis (Supplementary Figure S1). The electropherogram showed only one protein band with a mass of 150 kDa corresponding to the mass of intact human IgG. After reduction in disulfide bonds by dithiothreitol (DTT), only two protein bands in the region of 50 and 25 kDa were detected, corresponding to the masses of IgG heavy and light chains, respectively. Thus, it was confirmed that IgG samples did not contain impurities of other proteins. In the next step, the IgG samples were tested for catalytic activity.

### 2.3. IgG-Dependent Hydrolysis of DNA

IgG samples were primarily tested for DNA hydrolyzing activity since such antibody activity had been previously demonstrated [39,40].

**Figure 1:**
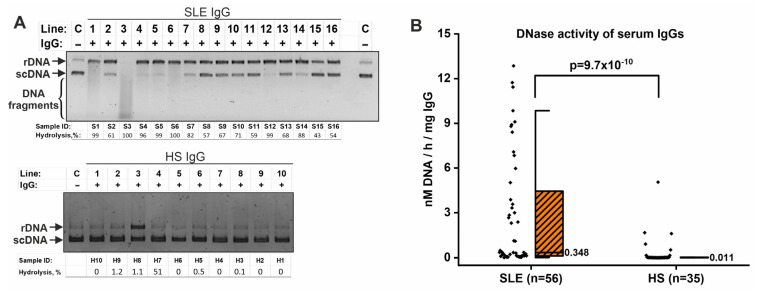
DNase activity of IgGs isolated from the serum of SLE patients and healthy subjects (HS). (**A**) Examples of the results of analysis of plasmid DNA hydrolysis products after 1 h incubation with IgG samples of SLE patients (top panel) and HS (bottom panel) by electrophoresis in 1% agarose gel. Plasmid DNA hydrolysis (%) was evaluated densitometrically by the transition from supercoiled (scDNA) to relaxed (rDNA) forms of plasmid DNA and shorter fragments. Complete conversion of DNA from scDNA to rDNA was taken as 100% hydrolysis. Lines C indicate control reactions without IgG. Lines marked with numbers indicate reactions with IgG samples. (**B**) Comparison of the level of specific DNase activity of IgG samples from SLE patients and healthy individuals. The black diamonds represent individual participants. Median values are indicated on boxplots. Statistical significance of differences was determined using the Mann–Whitney test.

Analysis of plasmid DNA hydrolysis showed that incubation of plasmid DNA with IgG samples of SLE patients resulted in DNA nicking (transition to the relaxed form from the supercoiled form) and DNA hydrolysis to smaller fragments (Figure 1A). Interestingly, the IgG samples of SLE patients varied significantly in activity level. Some IgG samples only resulted in the formation of relaxed DNA (e.g., samples S8–S11), while others hydrolyzed DNA to smaller fragments (e.g., sample S3) (Figure 1A). IgG from healthy individuals hydrolyzed DNA to a lesser extent, although some IgG samples exhibited fairly high activity (e.g., sample H7) (Figure 1A).

Densitometric analysis was used to quantitatively assess the level of IgG DNase activity (% DNA hydrolysis). An example of densitometric analysis is presented in Supplementary Figure S2. The data obtained were then expressed in units of specific DNase activity (raw data are presented in Supplementary Table S1). The level of specific DNase activity of SLE IgG samples (median value: 0.348 nM/h/mg IgG) was significantly higher (*p* = 9.7 × 10^−10^) than that of conditionally healthy donors (0.011 nM/h/mg IgG) (Figure 1B).

### 2.4. IgG-Dependent Hydrolysis of Histones

**Figure 2:**
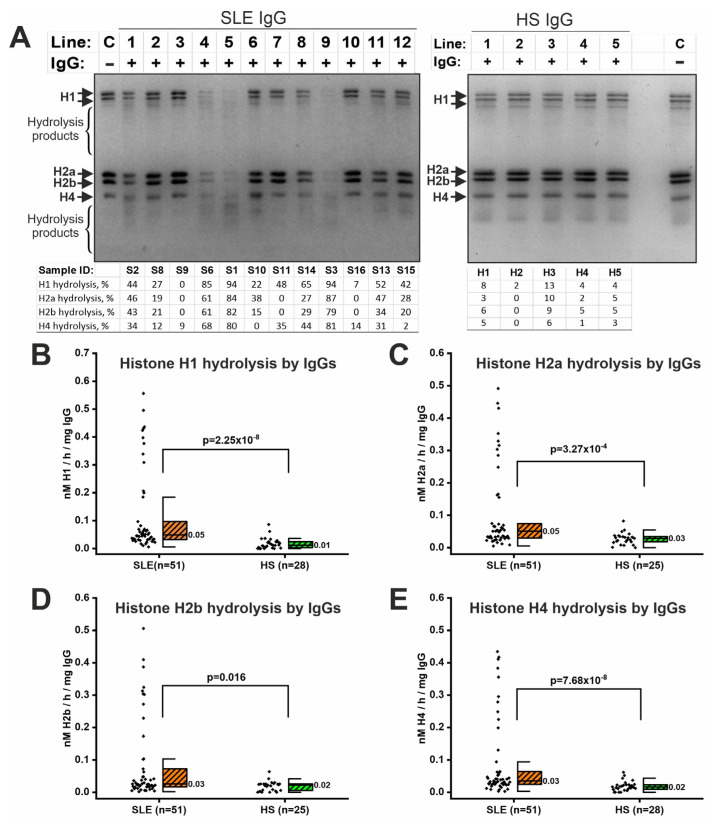
Histone-hydrolyzing activity of IgGs isolated from the serum of SLE patients and healthy subjects (HS). (**A**) Examples of the results of analysis of histone hydrolysis after 25 h incubation with IgG samples of SLE patients (left panel) and HS (right panel). Samples were separated by 15% SDS−PAGE and visualized by Coomassie staining. Bands corresponding to H1, H2a, H2b, and H4 histones and hydrolysis products are shown. Lines C indicate control reactions without IgG. Lines marked with numbers indicate reactions with IgG samples. The hydrolysis level (%) was calculated densitometrically in Image Lab 6.0 based on the decrease in intensity compared to the control. (**B**–**E**) Comparison of specific proteolytic activity in the hydrolysis of histones by IgG samples from SLE patients and healthy individuals. The black diamonds represent individual participants. Median values are indicated on boxplots. Statistical significance of differences was determined using the Mann–Whitney test.

IgG samples were also tested for their ability to hydrolyze histones, since these proteins are bound to DNA, and DNA-histone complexes can be antigens for autoantibody generation [59,60]. IgG samples from SLE patients have been shown to efficiently hydrolyze H1, H2a, H2b, and H4 histones (Figure 2A). Incubation of IgG samples resulted in a decrease in the intensity of histone bands and the appearance of shorter histone fragments (hydrolysis products). IgG samples from SLE patients differed in activity level, e.g., S1, S3, and S6 samples had high activity levels. IgG from healthy individuals generally had low histone-hydrolyzing activity (Figure 2A).

Densitometric analysis was used to quantify the histone-hydrolyzing activity of IgG (raw data are presented in Supplementary Table S1). The level of hydrolysis of H1, H2a, H2b, and H4 histones by antibodies of SLE patients was significantly higher than in healthy donors (*p* < 0.016) (Figure 2B). Thus, IgG samples from SLE patients efficiently hydrolyzed all four histones tested.

### 2.5. IgG-Dependent Hydrolysis of HMGB1

**Figure 3:**
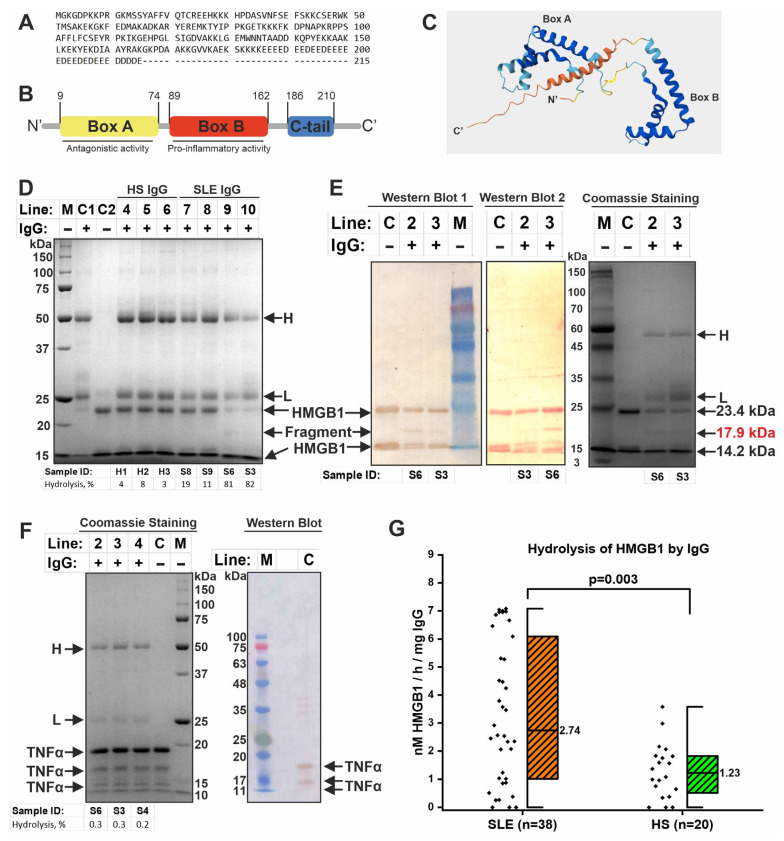
IgG of SLE patients hydrolyzes HMGB1 but not TNFα. (**A**) Sequence of recombinant HMGB1 used for analysis. (**B**) Schematic representation of the domain structure of full-length HMGB1. (**C**) Three-dimensional structure of full-length HMGB1 predicted by AlfaFold (identifier: AF-P09429-F1-v4). The average per-residue accuracy of the structure (pLDDT) score is represented by color: blue—very high, light blue—high, yellow—low, and brown—very low. (**D**) Examples of the results of analysis of HMGB1 hydrolysis after 17 h incubation with IgG samples from SLE patients (SLE IgG) and healthy subjects (HS IgG) by 15% SDS−PAGE and Coomassie staining. Line C1 indicates IgG without HMGB1. Line C2—control reactions without IgG. Lines marked with numbers—reactions with IgG samples. Lines M—molecular weight markers. The heavy (H) and light chain (L) of IgG, as well as HMGB1 with hydrolysis products, are visible. The hydrolysis level (%) was calculated densitometrically. (**E**) Analysis of HMGB1 hydrolysis products using Western blot with polyclonal anti-HMGB1 (left panel) or anti-His-Tag antibodies (middle panel) and Coomassie staining (right panel). Molecular masses of fragments were determined using Image Lab 6.0. The mass of the HMGB1 hydrolysis product is shown in red. Lines C—control reactions without IgG. Lines M—molecular weight markers. (**F**) Western blot (right panel) and 15% SDS−PAGE (left panel) analysis of TNFα hydrolysis after 17 h of incubation with IgG samples from SLE patients. Densitometric analysis revealed almost no hydrolysis. (**G**) Comparison of specific HMGB1-hydrolysing activity of IgG samples from SLE patients and healthy subjects. The black diamonds represent individual participants. Median values are indicated on boxplots. Statistical significance of differences was determined using the Mann–Whitney test.

HMGB1 is a non-histone protein that binds DNA and histones [20]. HMGB1 can also be an antigen for the formation of autoantibodies. Since IgG of SLE patients showed high levels of DNA- and histone-hydrolyzing activity in this study, we hypothesized that such antibodies may hydrolyze other DNA-associated proteins, including HMGB1. Recombinant HMGB1 representing the full-length (Met1~Glu215) protein with an additional N-terminal His-Tag was used to analyze the ability of IgG to hydrolyze HMGB1 (Figure 3A).

HMGB1 is known to consist of an A-box, a B-box, and an acidic C-terminal domain (Figure 3B,C). Recombinant HMGB1 migrated on the electrophoregram as two bands corresponding to the full-length protein (≈23.4 kDa) and probably a fragment without the A-box (≈14.2 kDa) (Figure 3D,E). Western blot analysis confirmed that the two initial bands observed represent fragments of HMGB1 (Figure 3E). Electrophoretic analysis with Coomassie staining showed that some IgG of SLE patients efficiently hydrolyze HMGB1, while IgG of healthy individuals has low proteolytic activity (Figure 3D). Some IgG samples from SLE patients had low proteolytic activity (e.g., samples S8 and S9), similar to samples from healthy individuals, while other samples hydrolyzed HMGB1 very efficiently (e.g., samples S6 and S3) (Figure 3D). Highly active IgG samples in the hydrolysis of HMGB1 also efficiently hydrolyzed DNA and histones (Figure 1A and Figure 2A). Additional examples of the results of analysis of HMGB1 hydrolysis by IgG samples are presented in Supplementary Figure S3. The level of HMGB1 hydrolysis in all samples was calculated densitometrically and converted into specific activity (raw data are presented in Supplementary Table S1). The results showed that IgG samples from SLE patients had 2-fold higher activity than samples from healthy individuals (Figure 3G).

Western blot analysis with HRP-linked polyclonal anti-HMGB1 antibody revealed a hydrolysis product of HMGB1 with a molecular mass of ≈17.9 kDa (Figure 3E, left panel). Such a hydrolysis product was observed in almost all IgG samples with high activity (*n* = 15). Western blot with anti-His-Tag antibody showed that the HMGB1 hydrolysis product (≈17.9 kDa) has an N-terminal His-Tag (Figure 3E, middle panel). The estimated molecular weight of the HMGB1 fragment consisting of A- and B-box should be approximately 19.5 kDa. Therefore, based on these data, the putative HMGB1 hydrolysis site was located in the B-box, but the specific hydrolysis site could not be determined. Other products with a mass of less than 15 kDa were also visible, but could not be resolved. Therefore, there may be other hydrolysis sites in other domains of HMGB1. Thus, hydrolysis of HMGB1 by IgG samples resulted in the formation of several products.

Since histones and HMGB1 are DNA-binding proteins, we decided to test the IgG-dependent hydrolysis of tumor necrosis factor-α (TNFα) as a control protein with other functions. TNFα is known to be a proinflammatory cytokine without DNA-binding properties [61]. It can also be present in the blood and is accessible to antibodies. Recombinant TNFα was used for analysis. TNFα migrated during electrophoresis as a main band (≈19.5 kDa) and two bands with lower mass (Figure 3F). Western blot confirmed that all observed bands represent fragments of TNFα. As a result, it was shown that IgG samples from SLE patients (*n* = 5) and healthy subjects (*n* = 5) did not hydrolyze recombinant TNFα (Figure 3F), although these samples effectively hydrolyzed DNA, histones, and HMGB1 (Figure 1A and Figure 2A).

Thus, IgG of SLE patients effectively hydrolyzed DNA and DNA-associated proteins (histones and HMGB1), but did not hydrolyze the control protein TNFα under the same conditions.

### 2.6. Catalase-like Activity of IgGs

**Figure 4:**
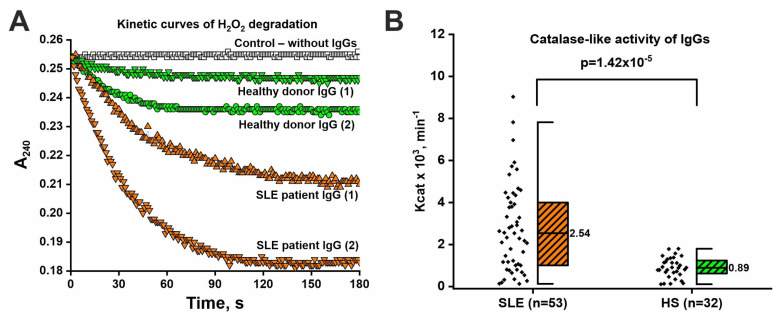
Catalase-like activity of IgGs isolated from the serum of SLE patients and healthy subjects (HS). (**A**) Example of kinetic curves of hydrogen peroxide degradation for two SLE patients and two HS. (**B**) Comparative analysis of IgGs catalase-like activity levels in SLE and HS groups. The black diamonds represent individual participants. Median values are indicated on boxplots. Statistical significance of differences was determined using the Mann–Whitney test.

For a more thorough analysis of the repertoire of catalytic antibodies in SLE, we also studied the catalase-like activity of IgG. We hypothesized that, in addition to IgG with hydrolytic activities, the general pool of antibodies may contain antibodies with other activities, including catalase-like activity. Prooxidative state and decreased activity of antioxidant enzymes, including catalase, are observed in SLE [62]. It has been previously shown that human antibodies may have redox activity [52], but the catalase-like activity of IgG in SLE has not yet been investigated. It has been shown that the addition of IgG samples to the reaction mixture resulted in a decrease in optical density caused by hydrogen peroxide decomposition (Figure 4A). Interestingly, catalase-like activity was exhibited by IgG samples from both SLE patients and healthy individuals. However, IgG samples of SLE patients showed about 2.5-fold higher levels of catalase-like activity than healthy individuals (Figure 4B). The raw data are presented in Supplementary Table S1. Thus, it was shown that SLE patients’ IgG possesses not only hydrolytic activities (hydrolyze DNA, histones, and HMGB1) but also catalase-like activity.

### 2.7. Verification That Catalytic Activity Belongs to Antibodies

A number of criteria have previously been developed to attribute catalytic activity to IgG [63]. These criteria include the following: (1) the use of affinity sorbents for IgG isolation; (2) the absence of catalytic activity after IgG sorption from the mixture; (3) differences in IgG activity levels between patients and healthy individuals, even though they are isolated using the same methods; (4) the observed kinetic parameters of hydrolysis differ significantly from those of classical enzymes; (5) the cleavage sites differ from sites characteristic of enzymes [63]. Using these criteria, numerous studies have previously confirmed that antibodies possess a number of catalytic activities [28,30,38]. In this work, one of the criteria was the use of affinity sorbents to isolate IgG. Secondly, zymographic analysis was used to identify proteins exhibiting enzymatic activity and definitively attribute catalytic activity to IgG [57] (Supplementary Figure S4). After SDS-PAGE of SLE patients’ IgG mix and staining with ethidium bromide, a decrease in DNA staining intensity occurred in areas with proteins with nuclease activity. It is shown that the position of areas of decreased DNA staining intensity corresponds to the position of the light chain and the dimer of two heavy chains, indicating that both light and heavy chains exhibit DNase activity. In addition, this experiment confirms the absence of DNase impurities (they differ in molecular mass, and if they were present, other bands would be detected). Thirdly, we also showed that sorption of IgG from the mixture on an affinity sorbent leads to a loss of catalytic activity (Supplementary Figure S5).

### 2.8. Patients’ Stratification Based on IgG DNase Activity Level

**Figure 5:**
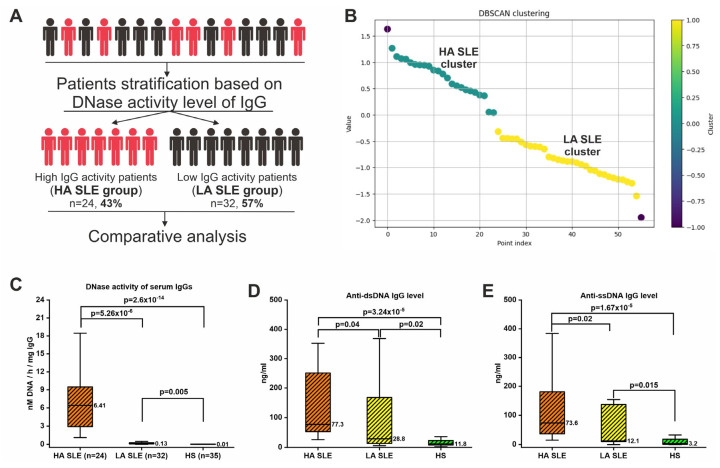
Stratification of SLE patients based on DNase activity level of IgGs and comparative analysis between groups. (**A**) Schematic representation of the stratification of patients into two groups: with high (HA SLE group) and low DNase activity of antibodies (LA SLE group). (**B**) Results of the DBSCAN algorithm used to stratify patients into two groups. (**C**) Comparison of IgG DNase activity level in HA SLE, LA SLE, and healthy subjects (HS) groups. (**D**,**E**) Comparison of the concentration of anti-dsDNA (**D**) and anti-ssDNA antibodies (**E**) in HA SLE, LA SLE, and HS groups. Statistical significance of differences was determined using the Kruskal–Wallis test.

Analysis of the catalytic activity of IgG samples revealed significant variation in activity level (Figure 1, Figure 2 and Figure 3), especially in the case of DNase activity (Figure 1). Therefore, for further analysis, SLE patients were divided into two groups: with high (HA SLE group) and low DNase activity of antibodies (LA SLE group) (Figure 5A). The density-based spatial clustering of applications with noise (DBSCAN) algorithm was applied for patient stratification (Figure 5B). The DBSCAN algorithm allows for density-based clustering and is often more suitable for handling complex data distributions than, for example, the K-means clustering algorithm. Analysis of the distribution histogram suggested a bimodal distribution, indicating the existence of two groups in the sample (Supplementary Figure S6). Using the DBSCAN algorithm, the threshold value was calculated (log10(y) = 0.04, consequently y = 10^0.04^ = 1.1 nM/h/mg IgG) based on which the sample of patients was divided into two groups (Figure 5B). In total, 24 patients (43%) were classified in the HA SLE group, and 32 patients (57%) were in the LA SLE group. The level of DNase activity of IgG samples in the HA SLE group was expectedly significantly (49 times) higher than that in the LA SLE group and in the control group (Figure 5C). Notably, the level of DNase activity of IgG in the LA SLE group was also higher than that in healthy individuals.

Interestingly, the concentration of anti-dsDNA and anti-ssDNA antibodies in the HA SLE group was more than two times higher than in the LA SLE group (Figure 5D,E). At the same time, the level of anti-DNA antibodies in the LA SLE group was also significantly higher than in conditionally healthy individuals (Figure 5D,E).

Comparison of the level of histone-hydrolyzing activity of IgG also revealed a significant increase in activity in the HA SLE group compared to the LA SLE group (Supplementary Figure S7A–D). The level of HMGB1-hydrolyzing activity was also higher in the HA SLE group (Figure S7E). Thus, the HA SLE group was characterized by more efficient hydrolysis of four histones and HMGB1. The level of proteolytic activity of IgG was also higher in the LA SLE group compared to the control, except for the hydrolysis of H2a and H2b histones (Figure S7B,C).

Remarkably, the level of IgG catalase-like activity was not significantly different between HA SLE and LA SLE groups (Figure S7F). Nevertheless, the level of IgG catalase-like activity was significantly higher in HA SLE and LA SLE groups compared with the group of healthy individuals (Figure S7F). Thus, the level of IgG catalase-like activity was consistently elevated in HA SLE and LA SLE groups.

### 2.9. Clinical Associations

Subgroups of patients with high/low IgG DNase activity levels could differ in clinical manifestations of the disease, so we compared clinical and anamnestic data in HA SLE and LA SLE groups (Table 2). The age of the patients did not differ between the groups. However, patients in the HA SLE group were characterized by shorter disease duration compared to the LA SLE group. SELENA-SLEDAI scores were not significantly different. Among the HA SLE group, there were more patients with a subacute and fewer patients with a chronic course of SLE. Thus, patients in the HA SLE group had shorter disease duration and mostly had subacute SLE.

Multiple regression analysis was performed to evaluate the effect of various factors on the patients’ condition, measured by SELENA-SLEDAI. The level of IgG DNase activity and disease duration were significant predictors of the SELENA-SLEDAI score (Table 3), with the determinants having a negative effect. Other variables, including anti-dsDNA antibody level, were not significant predictors.

Predictors of IgG DNase activity level were also studied (Table 4). IgG histone-hydrolyzing and catalase-like activity levels were predictors of IgG DNase activity level, with these predictors having a positive effect. In addition, the SELENA-SLEDAI score and disease duration were significant predictors of IgG DNase activity level. Notably, the t-statistic had a negative value, indicating a negative effect of these independent clinical predictors on activity level. The negative association of IgG DNase activity level with disease duration is also consistent with the results of subgroup analysis showing shorter disease duration in the HA SLE group (Table 2).

### 2.10. Correlation Analysis: The Levels of DNA-, Histone-, and HMGB1-Hydrolyzing Activity of IgG Are Closely Correlated

**Figure 6:**
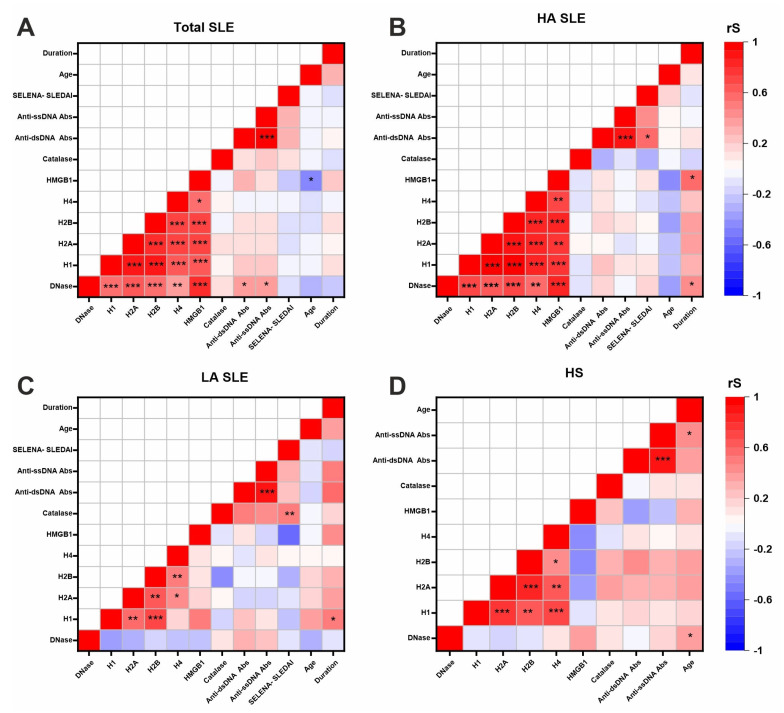
Correlations between serum IgG catalytic activity levels, clinical and anamnestic data in four study groups: total SLE (**A**), patients with high (HA SLE) (**B**) and low (LA SLE) (**C**) IgG DNase activity level, and healthy subjects (HS) (**D**). Abbreviations: DNase—IgG DNase activity level; H1, H2a, H2b, H4—IgG histone-hydrolyzing activity levels in the hydrolysis of H1, H2a, H2b, and H4, respectively; HMGB1—IgG HMGB1-hydrolyzing activity level; catalase—catalase-like activity; duration—disease duration. Rs—Spearman correlation coefficient. *—0.01 < *p* < 0.05; **—0.001 < *p* < 0.01; ***—*p* < 0.001.

Correlation analysis was performed in four groups: the total group of SLE patients, the HA SLE group, the LA SLE group, and the group of healthy subjects (Figure 6).

The most striking result of the correlation analysis was the close correlation between the level of DNA-, histone-, and HMGB1-hydrolyzing activity level of IgG, both in the total group of SLE patients (Figure 6A) and in the HA SLE group (Figure 6B). Moreover, the level of catalase-like activity did not correlate with the levels of DNase and proteolytic activity of antibodies. Notably, this correlation disappeared in the LA SLE group (Figure 6C). In addition, such a correlation was not observed in the group of healthy subjects (Figure 6D).

It was also shown that anti-dsDNA and anti-ssDNA antibody levels directly correlated with IgG DNase activity level (Rs = 0.40, *p* = 0.04, and Rs = 0.39, *p* = 0.03, respectively) in the total group of SLE patients (Figure 6A). Anti-dsDNA antibody levels were also directly correlated with SELENA-SLEDAI score (Rs = 0.59, *p* = 0.01) in the HA SLE group (Figure 6B). In the LA SLE group (Figure 6C), no correlation of anti-DNA antibody levels with SELENA-SLEDAI score was observed.

The level of DNA- and HMGB1-hydrolyzing activity of IgG was positively correlated with the disease duration (Rs = 0.43, *p* = 0.04, and Rs = 0.60, *p* = 0.01, respectively) in the HA SLE group (Figure 6B).

In the LA SLE group (Figure 6C), IgG catalase-like activity level was positively correlated with the SELENA-SLEDAI score (Rs = 0.53, *p* = 0.001). The level of IgG-dependent histone H1 hydrolysis was positively correlated with the disease duration (Rs = 0.48, *p* = 0.02).

In the group of healthy subjects (Figure 6D), only a positive correlation of IgG DNase activity level (Rs = 0.38, *p* = 0.04) and anti-ssDNA antibody levels (Rs = 0.46, *p* = 0.01) with the age of the participants was revealed.

## 3. Discussion

### 3.1. IgG-Dependent Hydrolysis of DNA and DNA-Associated Proteins in SLE

Antinuclear antibodies, including anti-dsDNA antibodies, represent a heterogeneous pool of autoantibodies with different origins, antigenic properties, and physicochemical features in SLE [59,64,65]. However, the triggering antigens and molecular mechanisms leading to the generation of antinuclear antibodies are not yet fully understood. Chromatin components, including complexes of DNA with DNA-associated proteins, are an important source of autoantigens for the formation of antinuclear antibodies in SLE [59,65,66,67]. Antigenic chromatin may be present in the form of nucleosome-associated soluble fragments, neutrophil extracellular traps, and/or as part of microparticles formed during apoptosis [65,68,69]. Such potentially antigenic complexes of DNA with DNA-associated proteins may trigger the formation of both classical high-affinity autoantibodies (including anti-dsDNA) [65,67,70] and catalytic antibodies [60].

This study investigated catalytic antibodies directed against DNA and DNA-associated proteins (histones and HMGB1). It is shown that antibodies from SLE patients hydrolyze DNA, histones, and HMGB1 more effectively than those from healthy individuals (Figure 1, Figure 2 and Figure 3). The results obtained about the high level of IgG DNA- and histone-hydrolyzing activity in SLE (Figure 1 and Figure 2) are consistent with previous findings [39,40,41,46]. This study is the first to identify HMGB1 hydrolysis by antibodies of SLE patients (Figure 3). At the same time, antibodies from SLE patients did not hydrolyze the control protein TNFα, which does not have DNA-binding properties (Figure 3F). Thus, this study shows that SLE antibodies hydrolyze both DNA and DNA-associated proteins such as histones and HMGB1. These data expand the knowledge of the repertoire of catalytic antibodies in SLE.

This work also demonstrated for the first time that the levels of DNA-, histone-, and HMGB1-hydrolyzing activity of IgG are closely correlated in SLE (Figure 6A), indicating cross-reactivity of autoantibodies in SLE. A growing body of research, including studies using monoclonal antibodies, shows that polyreactive or cross-reactive anti-DNA autoantibodies that recognize multiple antigens are generated in SLE [71,72,73]. There is evidence that anti-DNA antibodies cross-react with collagen, complement component 1q, ribosomal P proteins, N-methyl-d-aspartate receptor (NMDAR), and others [11]. For example, anti-DNA antibodies that cross-react with NMDAR mediate apoptotic death of neurons and are associated with neuropsychiatric SLE [73]. Another example of cross-reactive antibodies in SLE is antibodies to deoxyribonuclease 1 Like 3 (DNase1L3), the main enzyme for removing apoptotic material from the bloodstream and reducing chromatin immunogenicity [74,75]. It has been shown that anti-DNase1L3 antibody-dependent decrease in DNase1L3 activity is associated with autoreactivity to DNA and DNA-associated proteins in microparticles in sporadic SLE [75]. Moreover, there are examples of cross-reactive catalytic antibodies that hydrolyze DNA, histones, and other proteins in SLE [46,76]. Thus, the tight correlation between the level of IgG-dependent DNA and DNA-binding proteins hydrolysis identified in this study (Figure 6A) may be associated with the cross-reactivity of the catalytic antibodies formed. However, this assumption requires experimental validation in further studies. On the other hand, class or isotype switch and somatic hypermutations of the variable chain of IgG may also affect the catalytic activity of antibodies and possibly contribute to polyreactivity [77].

### 3.2. Associations with Clinical Symptoms

This study, through a relatively large sample size, showed that patients differed significantly in the level of IgG hydrolytic activities, especially DNase activity (Figure 4), and that the level of activity may be clinically related. After stratifying patients using the DBSCAN algorithm into two groups with high and low IgG DNase activity, a 49-fold difference in median activity values was identified (Figure 5). Patients exhibiting elevated IgG DNase activity demonstrated a reduced disease duration (*p* = 0.03), a higher incidence of subacute course (*p* = 0.048), and a tendency towards increased disease exacerbation (*p* = 0.08) (Table 2). Patients with high IgG DNase activity also had higher levels of canonical anti-DNA antibodies (Figure 5D,E). Moreover, IgG DNase activity level, along with disease duration, was found to be a significant predictor of SELENA-SLEDAI score (Table 3), while anti-DNA antibody level was not a significant predictor. However, regression models explained only a small portion of the variability. Thus, IgG with DNase activity may be associated with clinical manifestations of SLE, but further research is needed to clarify the catalytic antibody-related molecular mechanisms leading to clinical symptoms.

IgG DNase activity level was also positively correlated with the level of canonical anti-DNA antibodies (Figure 6A), indicating that both high-affinity and catalytic antibodies may be formed during antibody formation in response to the antigen.

### 3.3. Catalase-like Activity of IgG in SLE

This work is the first to reveal high catalase-like activity of antibodies in SLE. The mechanisms of antibody formation with redox activity are still poorly understood, but it is assumed that they differ from the mechanisms of anti-DNA antibody formation. The formation of such catalytic antibodies may be associated with the production of autoantibodies to catalase and superoxide dismutase, which have been identified in SLE [78]. Therefore, this study also investigated catalase-like activity in IgG samples from patients who exhibited DNase activity and proteolytic activity. Remarkably, the level of IgG catalase activity did not correlate with the hydrolytic activities (DNA-, histone-, and HMGB1-hydrolyzing), indicating differences in the mechanisms of formation of such antibodies. Additionally, in contrast to DNase activity and histone-hydrolyzing activity, for catalase-like activity of IgG, no marked division of patients into two groups according to this activity was demonstrated.

The high level of catalase-like activity of IgG may be associated with the disturbance of redox homeostasis in SLE [62]. For example, a decreased ratio of reduced glutathione (GSH) to oxidized glutathione (GSSG) was found in patients with SLE, which correlated with the disease activity index and the degree of organ damage [79]. A decrease in the activity of antioxidant enzymes such as superoxide dismutase, glutathione peroxidase, and catalase has also been shown [62]. Oxidative modification of self-antigens triggers autoimmunity and promotes disease progression [80]. Hyperproduction of hydrogen peroxide is known to be one of the reasons for the increased apoptosis and impaired phagocytosis observed in SLE [56]. Mitochondrial dysfunction in T cells contributes to the release of highly diffusible inflammatory lipid hydroperoxides in SLE [80]. Since there is a decrease in the activity of glutathione peroxidase family proteins in SLE [81], it is possible that antibodies with catalase activity are included in the process of ROS detoxification, complementing the action of specialized antioxidant defense enzymes. However, the contribution of antibodies with catalase-like activity to antioxidant defense in SLE remains unclear.

### 3.4. The Possible Role of Catalytic Antibodies in SLE

There is still no consensus in the literature regarding the role of catalytic antibodies in autoimmune pathologies [30]. The results of this study cannot establish the role of catalytic antibodies in SLE. On the one hand, antibodies that hydrolyze DNA and DNA-associated proteins may play a positive role, since hydrolysis of such antigens may reduce the immune response. However, this assumption requires experimental validation. On the other hand, antibodies can play a negative role by hydrolyzing functional molecules. For example, there is evidence that catalytic antibodies in arrhythmogenic cardiomyopathy patients cleave intercalated disk proteins (N-cadherin and desmoglein 2), leading to a decrease in cardiomyocyte cohesion [82]. In addition, the effect of catalytic antibodies on complement activation and Fc receptor binding has not yet been studied. Thus, further studies are needed to clarify the role of DNase and proteolytic (histone- and HMGB1-hydrolyzing) antibodies in SLE.

### 3.5. Limitations

The results of this study should be interpreted with caution. First, this is an observational study, so the clinical associations of IgG catalytic activity identified do not prove a causal relationship. Second, the assumption about the protective implications of catalytic IgG needs biological validation. Therefore, functional in vivo studies are needed to demonstrate the effects of catalytic antibodies in removing proinflammatory molecules (DNA, histones, HMGB1, etc.).

## 4. Materials and Methods

### 4.1. Patients

The protocol of this study was approved by the Local Ethics Committee of the Institute of Chemical Biology and Fundamental Medicine (protocol No. 3, from 19 June 2023). A total of 56 patients with SLE were selected for the study. The selection of patients and the verification of the diagnosis were carried out by experienced rheumatologists at the Department of Rheumatology, Immunopathology Clinic, Research Institute of Fundamental and Clinical Immunology (Novosibirsk, Russia). The inclusion criteria for patients were as follows: consent to participate in the study; diagnosis of SLE (M32, ICD-10) in accordance with the Russian Association of Rheumatologists and EULAR recommendations [83]; age from 18 to 75 years; absence of oncological diseases, psychiatric diseases, and acute infectious diseases at least one month before the study; no vaccinations at least one month before the study; no trauma at least three months before the study; no pregnancy or childbirth at least one year before the study. The SELENA-SLEDAI (Safety of Estrogens in Lupus Erythematosus National Assessment–SLE Disease Activity Index) scale was applied to assess SLE disease activity [84]. Each SLE patient received at least two immunotropic drugs, one of which was a corticosteroid (1 to 20 mg/day depending on the drug). The most commonly used nonsteroidal drugs were methotrexate, mycophenolate mofetil, celecoxib, hydroxychloroquine, and tenoxicam.

In addition to patients with SLE, 35 conditionally healthy donors were included in the study. The inclusion criteria for healthy subjects were as follows: consent to participate in the study; age 18–75 years; absence of rheumatologic, oncologic, and endocrinologic diseases at the time of the study and in the history; absence of psychiatric diseases; absence of acute infectious diseases at least one month before the study; no vaccinations at least one month before the study; no trauma at least three months before the study; no pregnancy and childbirth at least one year before the study.

### 4.2. Isolation and Characterization of IgG Samples from Serum

The participants’ blood was collected in vacuum tubes with silicon dioxide (Shandong Medical Products Factory, Jinan, China) and centrifuged (2000× *g*, 15 min) to obtain the serum. Samples of polyclonal IgG were isolated from the serum by affinity chromatography using an ÄKTA Start chromatography system (GE Healthcare, Chicago, IL, USA) in accordance with a previously developed protocol [49,57,58]. Briefly, the serum was centrifuged (7500× *g*, 5 min), diluted in a 1:3 ratio with buffer A (50 mM Tris-HCl, pH 7.5 + 150 mM NaCl), and applied to a column (rProtein G Seplife 4FF (Sunresin New Materials Co., Ltd., Xi’an, China), 1 mL). All irrelevant impurities were removed by washing the column with buffer A (8 column volumes), 1% Triton solution in buffer A (3 column volumes), and buffer A (15 column volumes). IgG was eluted with 100 mM Gly-HCl, pH 2.6. The obtained IgG samples were additionally processed by HPLC gel filtration using a Superdex 200 HR 10/30 column (Cytiva, Uppsala, Sweden), neutralized with an alkaline buffer (1 M Tris-HCl, pH 8.8), and dialyzed in 50 mM Tris-HCl, pH 7.5. IgG concentration was then determined spectrophotometrically using a BioSpectrometer Kinetic (Eppendorf, Hamburg, Germany). The homogeneity of the isolated IgG samples was checked using gradient denaturing SDS-PAGE (4–18%), followed by staining with 0.01% Coomassie R-250 without and with the addition of a disulfide bond reducing agent, DTT, to the samples before applying them to the gel. After staining, the result was recorded in a Gel Doc XR+ gel documentation system (Bio-Rad, Hercules, CA, USA).

### 4.3. IgG-Dependent DNA-Hydrolyzing Activity Assay

The level of IgG DNase activity was determined by agarose gel electrophoresis using pBluescript plasmid DNA (4588 base pairs, molecular mass 2.95 kDa) according to the methodology used previously [57]. The reaction mixture (10 μL) containing 20 mM Tris-HCl, pH = 7.5, 18 μg/mL pBluescript plasmid DNA, 5 mM MgCl_2_, and 0.1 mg/mL IgG or equivalent buffer volume in case of control was incubated at 37 °C for 30 min. Electrophoresis in 1% agarose gel (150 mA, 80 V) was performed to separate the hydrolysis products. The gel was then stained with ethidium bromide and documented using a Gel Doc XR+ gel documentation system (Bio-Rad, Hercules, CA, USA). Densitometric analysis of electrophoregrams was performed in Image Lab 6.0 (Bio-Rad, Berkeley, CA, USA). The level of IgG DNase activity was evaluated densitometrically by the transition of plasmid DNA from supercoiled to relaxed form and shorter forms of DNA (for more information, see Supplementary Figure S2). The results were presented as nmol DNA/1 h (incubation time)/1 mg IgG.

### 4.4. Zymographic (In Situ) Analysis of DNA-Hydrolyzing Activity of IgG Samples

Zymographic analysis was performed as previously described to identify in situ proteins exhibiting nuclease activity [57]. A mix of IgG samples of SLE patients (20 μg protein/line) was separated in a 4–18% gradient SDS-PAGE containing copolymerized calf thymus polymeric DNA (cat # D4522, Merck, Darmstadt, Germany). After washing with SDS in 20 mM Tris–HCl (pH 7.5) and incubation for 48 h in buffer (20 mM Tris–HCl (pH 7.5), 4 mM MgCl_2_, and 0.2 mM CaCl_2_) under optimal conditions for hydrolysis, the gel was stained with ethidium bromide. The same gel was then stained with Coomassie R-250 to visualize proteins using a Gel Doc XR+ gel documentation system (Bio-Rad, Hercules, CA, USA).

### 4.5. IgG-Dependent Histone-Hydrolyzing Activity Assay

The level of IgG-dependent histone-hydrolyzing activity was determined by Polyacrylamide Gel Electrophoresis using lyophilized histones type II-A (mixture of H1, H2a, H2b, H3, and H4 histones; cat # H9250, Merck, Darmstadt, Germany) according to previously described methodology [58]. The reaction mixture (10 μL) contained 20 mM Tris-HCl (pH 7.5), 1 mg/mL histone mixture, and 0.1 mg/mL IgG or equivalent buffer volume in the case of control. The reaction mixtures were incubated for 25 h at 37 °C. The reaction was stopped with a stop buffer containing 50 mm Tris-HCl (pH 6.8), 10% glycerol, 2% sodium dodecyl sulfate, 0.025% bromophenol blue, followed by freezing at −70 °C before analysis. To visualize the hydrolysis products, electrophoresis was performed in a 15% polyacrylamide gel (acrylamide to bisacrylamide ratio: 32:1) followed by Coomassie R-250 staining. Electrophoregrams were obtained using a Gel Doc XR+ gel documentation system (Bio-Rad, Hercules, CA, USA). The level of histone-hydrolyzing activity of IgG was determined densitometrically by a decrease in intensity of the protein bands of each of the histones using Image Lab 6.0 (Bio-Rad, Berkeley, CA, USA). The result was presented as nmol histone/1 h (incubation time)/1 mg IgG.

### 4.6. IgG-Dependent HMGB1-Hydrolyzing Activity Assay

Commercial recombinant HMGB1 (cat # RPA399Hu01, Cloud-Clone Corp., Katy, TX, USA) representing the full-length protein (Met1~Glu215) with an additional N-terminal His-Tag was used to analyze the ability of IgG to hydrolyze HMGB1. The reaction mixture (10 μL) contained 20 mM Tris-HCl (pH 7.5), 0.12 mg/mL HMGB1, and 0.1 mg/mL IgG or equivalent buffer volume in the case of control. To stop the reaction after incubation for 17 h at 37 °C, a reducing stop buffer containing 50 mM Tris-HCl (pH 6.8), 100 mM dithiothreitol, 10% glycerol, 2% sodium dodecyl sulfate, and 0.025% bromophenol blue was added to the reaction mixture, followed by freezing at −70 °C before analysis. Analysis of hydrolysis products was performed using reducing 10% SDS-PAGE followed by Coomassie R-250 staining. The hydrolysis products were also visualized by Western blot. In the first case, after transferring the proteins to a polyvinylidene fluoride membrane, blocking with a buffer containing bovine serum albumin, and washing, HMGB1 was stained with rabbit HRP-linked polyclonal anti-HMGB1 antibody at a dilution of 1:2150 (cat # LAA399Hu91, Cloud-Clone Corp., Katy, TX, USA). In the second case, mouse anti-His-Tag antibody conjugated with alkaline phosphatase at a dilution of 1:20,000 (clone: AD1.1.10, isotype: IgG1, cat # MCA1396A, Bio-Rad Laboratories, Inc., Hercules, CA, USA) was used for Western blotting. To stain the blot, a mixture of two dyes [5-bromo-4-chloro-3-indolyl-phosphate (BCIP, #R0822, Thermo Fisher Scientific GmbH, Dreieich, Germany) and nitro blue tetrazolium (NBT, #N6495, Thermo Fisher Scientific GmbH, Dreieich, Germany)] was used in alkaline phosphatase buffer (100 mM Tris-HCl, pH 9.5, 100 mM NaCl, and 10 mM MgCl_2_). The results were recorded in the Gel Doc XR+ gel documentation system (Bio-Rad, Hercules, CA, USA). The level of HMGB1-hydrolyzing activity of IgG was determined densitometrically by a decrease in the intensity of the protein bands of HMGB1 using Image Lab 6.0 (Bio-Rad, Berkeley, CA, USA). The result was presented as nmol HMGB1/1 h/1 mg IgG.

To represent the spatial structure of HMGB1, data from AlphaFold DB version 2022-11-01 (identifier: AF-P09429-F1-v4), created with the AlphaFold Monomer v2.0, were used [85]. AlphaFold data were used because the UniProt database currently does not have a resolved structure for the full-length HMGB1. The average per-residue accuracy of the structure (pLDDT) score corresponding to the prediction confidence for HMGB1 was 76.12 (High).

### 4.7. Analysis of TNFα Hydrolysis by Antibodies

Recombinant active TNFα (cat # APA133Hu01, Cloud-Clone Corp., Katy, TX, USA) was used to analyze the ability of IgG to hydrolyze TNFα. The reaction mixture (10 μL) containing 20 mM Tris-HCl (pH 7.5), 0.12 mg/mL TNFα, and 0.1 mg/mL IgG or equivalent buffer volume in case of control was incubated for 17 h at 37 °C. The reaction was stopped with a reducing stop buffer and visualized using reducing 10% SDS-PAGE followed by Coomassie R-250 staining or by Western blotting as described previously (Section 4.6). For Western blotting, rabbit HRP-linked polyclonal antibody to TNFα at a dilution of 1:2000 (cat # LAA133Hu91, Cloud-Clone Corp., Katy, TX, USA) was used.

### 4.8. IgG-Dependent Catalase-like Activity Assay

The level of IgG catalase-like activity was determined by the rate of decomposition of hydrogen peroxide as a substrate using the spectrophotometric method, as described in [86]. The reaction mixture contained 30 mM H_2_O_2_, 50 mM potassium phosphate buffer, pH 7.0, and 0.1 mg/mL IgG. The decomposition of hydrogen peroxide was recorded by the decrease in optical density (λ = 240 nm) on a Genesis 10S Bio UV/Vis spectrophotometer (Thermo Fisher Scientific, Waltham, MA, USA) for 5 min at 25 °C. The level of IgG catalase-like activity was calculated according to [50], by determining the *k*_cat_ value for each IgG preparation using the formula *k*_cat_ = V (M/min)/[IgG] (M), where V represented the initial reaction rate of hydrogen peroxide decomposition and [IgG] was the total IgG concentration in the reaction mixture. The initial reaction rate was calculated from the slope of the linear section of the hydrogen peroxide decomposition kinetic curve. The value of H_2_O_2_ molar extinction coefficient ε = 0.081 mM^−1^ cm^−1^ was used in calculations [87].

### 4.9. Analysis of Catalytic Activity of Fractions After IgG Sorption from the Mixture

To prove that catalytic activity belongs to antibodies, an analysis of catalytic activity in fractions after IgG sorption on an affinity sorbent was performed (Supplementary Figure S5). An equimolar mixture of two IgG samples of SLE patients showing high activity in the hydrolysis of DNA and histones (430 μL, [IgG] = 1.5 mg/mL) was applied to a rProtein G Seplife 4FF (Sunresin New Materials Co., Ltd., Xi’an, China) column (1 mL) pre-equilibrated with TBS (50 mM Tris-HCl pH 7.5 + 150 mM NaCl). The flow-through fraction (fraction 1, 1 mL) containing unbound proteins was collected. The column was then washed with TBS (10 mL), and IgG was eluted with 100 mM Gly-HCl pH 2.6 (fraction 2, 1 mL). The fractions obtained were neutralized with 1 M Tris-HCl, pH 8.8, and dialyzed in 50 mM Tris-HCl, pH 7.5. A sample of an equimolar mixture of IgG prior to chromatography, fractions 1 and 2, was used to test DNase activity and histone-hydrolyzing activity as described above.

### 4.10. Determination of Anti-DNA IgG Concentration

The concentration of anti-dsDNA IgG was determined by ELISA using Vecto-dsDNA-IgG kit 12x8 A-8656 (Vector-Best, Novosibirsk, Russia). The concentration of anti-ssDNA IgG was determined by ELISA using the Vecto-ssDNA-IgG 12x8 A-8658 kit (Vector-Best, Novosibirsk, Russia). Optical density was measured on a Multiskan™ FC Microplate Photometer (Thermo Fisher Scientific GmbH, Dreieich, Germany) at a wavelength of 450 nm, according to the kit manufacturer’s instructions.

### 4.11. Statistical Processing and Visualization of Data

Statistical processing and visualization of data were performed in OriginPro 2021 software (OriginLab Corporation, Northampton, MA, USA). The distribution of data in the samples was determined using the Shapiro–Wilk test. Comparison of median values in two samples was performed using the Mann–Whitney test. Comparison of median values in three or more samples was carried out using the Kruskal-Wallis test and Dunn’s post hoc analysis. Differences in categorical features were assessed using the Chi-square test. Correlations were evaluated using Spearman’s criterion. Results with *p*-value < 0.05 were considered statistically significant.

Stratification of patients into two groups based on the level of DNAase activity of antibodies was performed using the density-based spatial clustering of applications with noise (DBSCAN) algorithm. The data were preliminarily normalized by log10 transformation. The analysis of data distribution and probability density function was performed using the Kernel Density Estimation (KDE) algorithm and distribution diagrams. The Jupyter Notebook interactive computing environment: https://jupyter.org/try-jupyter/notebooks/?path=notebooks/Intro.ipynb (accessed on 20 August 2025), utilizing NumPy 2.3.3, SciPy 1.16.2, Seaborn 0.13.2, and Matplotlib 3.10.6 Python libraries for computation and visualization of results, was used. Multiple linear regression to find independent variables for the dependent variables SELENA-SLEDAI and IgG DNAase activity level was performed in Jupyter Notebook version 5.2.2 using the above-mentioned libraries. Missing values in the independent variables were replaced by median values. The code used can be found on GitHub: https://github.com/Marken94/DNAse-activity-SLE/tree/main (accessed on 20 August 2025).

## 5. Conclusions

This study showed that antibodies in SLE patients effectively hydrolyze DNA and DNA-associated molecules such as histones and HMGB1. Thus, in SLE, not only are classic high-affinity antinuclear antibodies formed, but catalytic antibodies are also capable of hydrolyzing DNA and DNA-bound proteins. In addition, high catalase-like activity of IgG in SLE patients was detected. These results open up prospects for further investigation of the biological or pathological role of such antibodies in SLE.

## Figures and Tables

**Table 1:** Clinical and anamnestic data of SLE patients and healthy subjects.

Parameter	SLE (*n* = 56)	HS (*n* = 35)	*p*-Value
Sex (F/M, %)	100/0	100/0	-
Age, years	50.5 (40, 59)	45 (38, 59)	0.35
SLE duration, years	8.5 (4, 17)	-	-
SELENA-SLEDAI, score	6 (4, 10)	-	-
Anti-ssDNA IgG, ng/mL	39.6 (12, 154)	3 (1, 17)	0.00003
Anti-dsDNA IgG, ng/mL	61.5 (28, 189)	12 (8, 21)	0.00006
Course (acute/subacute/chronic, %)	2/27/71	-	-
SLE activity (high/moderate/low/minimal, %)	16/49/2/33	-	-
Phase (active/inactive, %)	96/4	-	-

Note: Results are presented as median value (Q1, Q3) for quantitative parameters or as relative frequencies (%) for categorical data. Mann–Whitney test or Chi-square test was used to assess the significance of differences. Abbreviations: SLE—systemic lupus erythematosus; HS—healthy subjects, SELENA-SLEDAI—Safety of Estrogens in Lupus Erythematosus: National Assessment–Systemic Lupus Erythematosus Disease Activity Index.

**Table 2:** Clinical characteristics of SLE patients in high (HA SLE) and low (LA SLE) IgG DNase activity groups.

Parameter	HA SLE (*n* = 24)	LA SLE (*n* = 32)	*p*-Value
Age, years	48 (41, 58)	53 (41, 61)	0.62
Disease duration, years	6 (3, 14)	12 (7, 20)	**0.03**
SELENA-SLEDAI, score	6 (4, 8)	8 (5, 10)	0.33
Course (chronic/subacute, %)	17%/83%	42%/58%	**0.048**
SLE activity (high/moderate/low/minimal, %)	8%/50%/0%/42%	21%/36%/4%/39%	0.73
Exacerbation (yes/no), %	92%/8%	71%/29%	0.08

Note: Results are presented as median value (Q1, Q3) for quantitative parameters or as relative frequencies (%) for categorical data. Significant differences (*p* < 0.05) calculated using Mann–Whitney test or Chi-square test are highlighted in bold.

**Table 3:** Predictors of SELENA-SLEDAI score in multiple regression analysis.

Variable	β	Standard Error of β	*t*-Statistic	*p*-Value	Adjusted R^2^
IgG DNase activity	−0.147	0.060	−2.458	**0.019**	0.092
IgG H1-hydrolysing activity	0.146	0.118	1.236	0.224	
IgG catalase-like activity	0.015	0.106	0.140	0.889	
Anti-dsDNA Abs level	0.187	0.117	1.599	0.118	
Age	−0.026	0.310	−0.083	0.934	
Disease duration	−0.190	0.087	−2.189	**0.035**	

Note: Significant differences (*p* < 0.05) are highlighted in bold.

**Table 4:** Predictors of IgG DNAse activity level in multiple regression analysis.

Variable	β	Standard Error of β	*t*-Statistic	*p*-Value	Adjusted R^2^
IgG H1-hydrolysing activity	1.129	0.214	5.267	**0.0001**	0.446
IgG catalase-like activity	0.252	0.209	1.209	0.232	
Anti-dsDNA Abs level	0.604	0.281	2.152	**0.036**	
SELENA-SLEDAI	−1.027	0.397	−2.585	**0.013**	
Age	−0.840	0.803	−1.046	0.301	
Disease duration	−0.504	0.212	−2.371	**0.022**	

Note: Significant differences (*p* < 0.05) are highlighted in bold.

## Data Availability

The main data supporting the findings of this study are available within the article/Supplementary Material. Raw data are available from the corresponding author upon reasonable request.

## Supplementary Materials

The following supporting information can be downloaded at: https://www.mdpi.com/article/10.3390/ijms26199635/s1.

## Abbreviations

The following abbreviations are used in this manuscript:

SLE	Systemic lupus erythematosus
DNase1L3	Deoxyribonuclease 1 Like 3
DBSCAN	The density-based spatial clustering of applications with noise algorithm
KDE	Kernel Density Estimation algorithm
dsDNA	Double-stranded DNA
ssDNA	Single-stranded DNA
scDNA	Supercoiled DNA
rDNA	Relaxed DNA
SELENA-SLEDAI	Safety of Estrogens in Lupus Erythematosus National Assessment—Systemic Lupus Erythematosus Disease Activity Index
HA SLE	High-activity SLE group
LA SLE	Low-activity SLE group
HMGB1	High-mobility group protein B1
NMDAR	N-methyl-d-aspartate receptor
HS	Healthy subjects
HPLC	High-Performance Liquid Chromatography
DTT	Dithiothreitol
