# Supplementary material for: Catalytic IgG Antibodies Hydrolyze DNA, Histones, and HMGB1 in Systemic Lupus Erythematosus
Source: Int J Mol Sci. 2025 Oct 2;26(19):9635. doi: 10.3390/ijms26199635 (PMC12524583; doi:10.3390/ijms26199635)
Supplement: Supplementary file 1 [file ijms-26-09635-s001.zip › ijms-3871899-supplementary.pdf]

Supplementary materials

Article

Catalytic IgG antibodies hydrolyze DNA, histones, and HMGB1 in systemic lupus erythematosus

Mark M. Melamud [1], Evgeny A. Ermakov [1, 2], Anna S. Tolmacheva [1], Irina A Kostrikina [1], Alexey E. Sizikov [1, 3], Georgy A. Nevinsky [1, 2] and Valentina N. Buneva [1, 2, *]

[1] Institute of Chemical Biology and Fundamental Medicine, Siberian Branch of the Russian Academy of Sciences, 630090, Novosibirsk, Russia
[2] Department of Natural Sciences, Novosibirsk State University, 630090, Novosibirsk, Russia
[3] Department of Rheumatology, Immunopathology Clinic, Research Institute of Fundamental and Clinical Immunology, Siberian Branch of the Russian Academy of Sciences, Novosibirsk, Russia
* Correspondence: buneva@niboch.nsc.ru (V.N.B.)

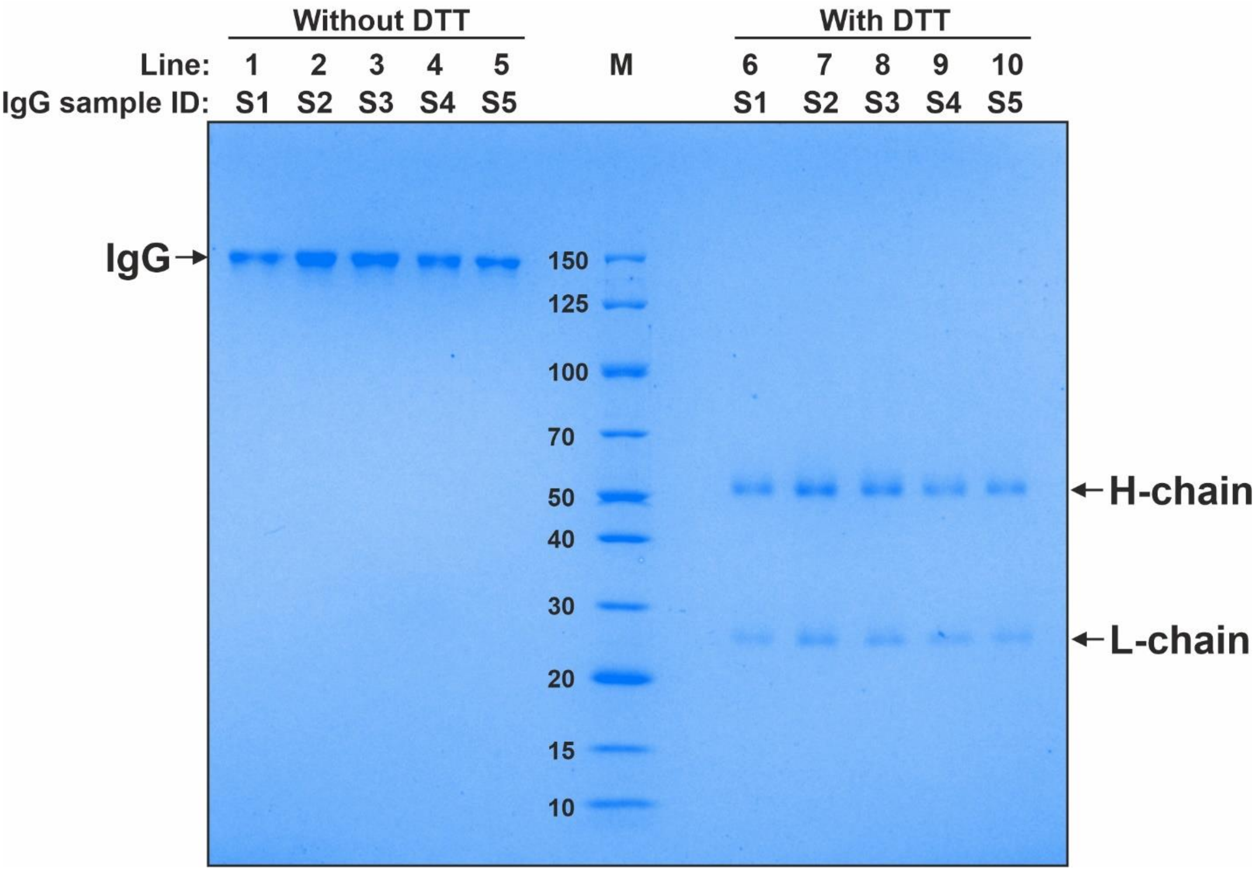

Figure S1. SDS-PAGE analysis of homogeneity of isolated IgG samples. IgG samples of SLE patients (15 µg protein/line) were separated in 4-18% gradient SDS-PAGE and stained with Coomassie-G-250. Lines 1-5: IgG samples without the disulfide bond-reducing agent (dithiothreitol, DTT) at a concentration of 10 mM; Lines 6-10: IgG samples with DTT. M – Protein molecular mass markers.

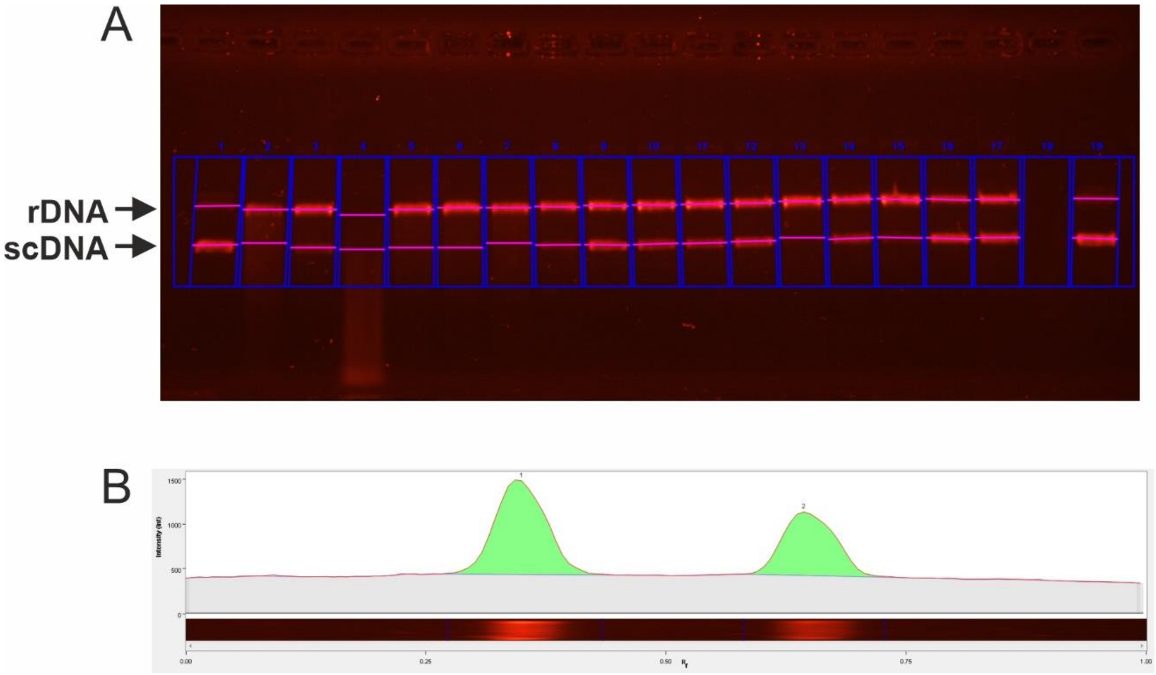

Figure S2. An example of densitometric analysis for determining IgG DNase activity. (A) The results of the analysis of plasmid DNA hydrolysis products after 1 hour of incubation with IgG samples from SLE patients using electrophoresis in 1% agarose gel (Fig. 1A in the manuscript) are presented. Plasmid DNA hydrolysis was detected by the transition of supercoiled (scDNA) to relaxed (rDNA) forms of plasmid DNA and shorter fragments. The electropherogram was analyzed using Image Lab 6.0. This program automatically detected the lines (highlighted in blue) and bands (highlighted in pink) of scDNA and rDNA plasmid DNA. (B) Line 12 (IgG sample S10) is shown as an example. The background (highlighted in gray) was subtracted for each line. The adjusted volume intensity for each band (highlighted in green) was then calculated. Finally, the IgG DNase activity level (% hydrolysis of plasmid DNA) for a specific IgG preparation was determined by the change in the adjusted volume intensity of the scDNA and rDNA bands of plasmid DNA. Complete conversion of DNA from scDNA to rDNA was taken as 100% hydrolysis. The results obtained were then normalized and presented as nmol DNA/1 h/1 mg IgG.

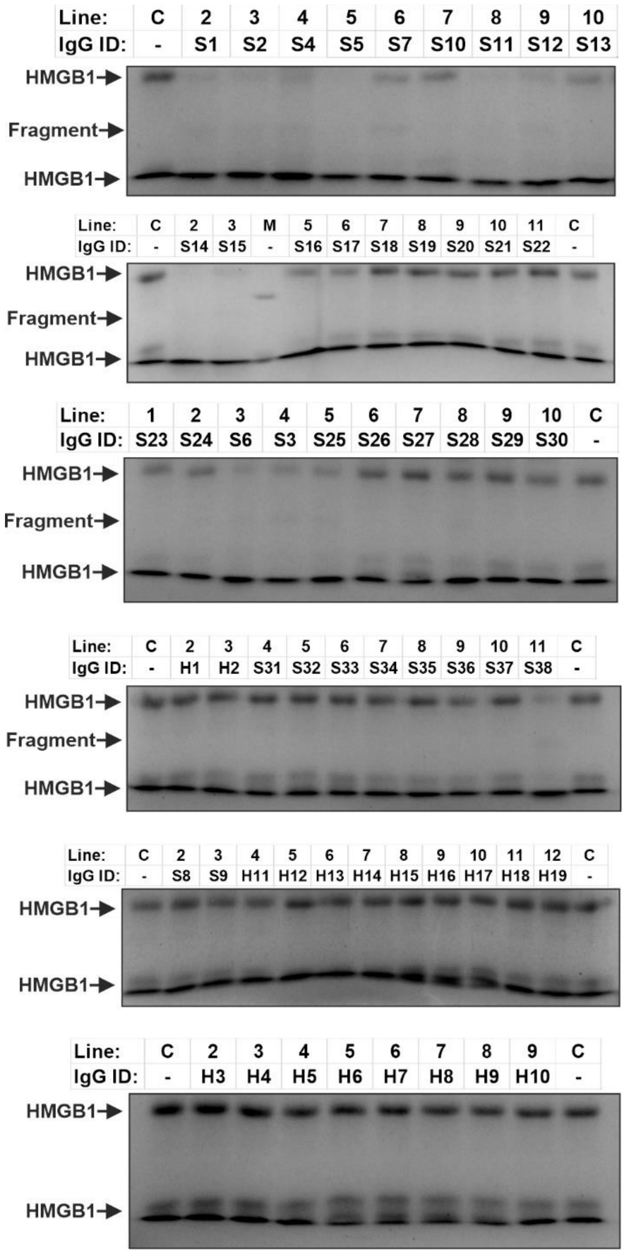

Figure S3. Additional examples of the results of analysis of HMGB1 hydrolysis by IgG samples of SLE patients and healthy subjects. Samples were separated by 15% SDS–PAGE and visualized by Coomassie staining. Lines C – control reactions without IgG. Lines M – molecular weight markers. Lines marked with numbers – reactions with IgG samples. IgG ID starting with S denotes samples of SLE patients and with H – samples of healthy subjects.

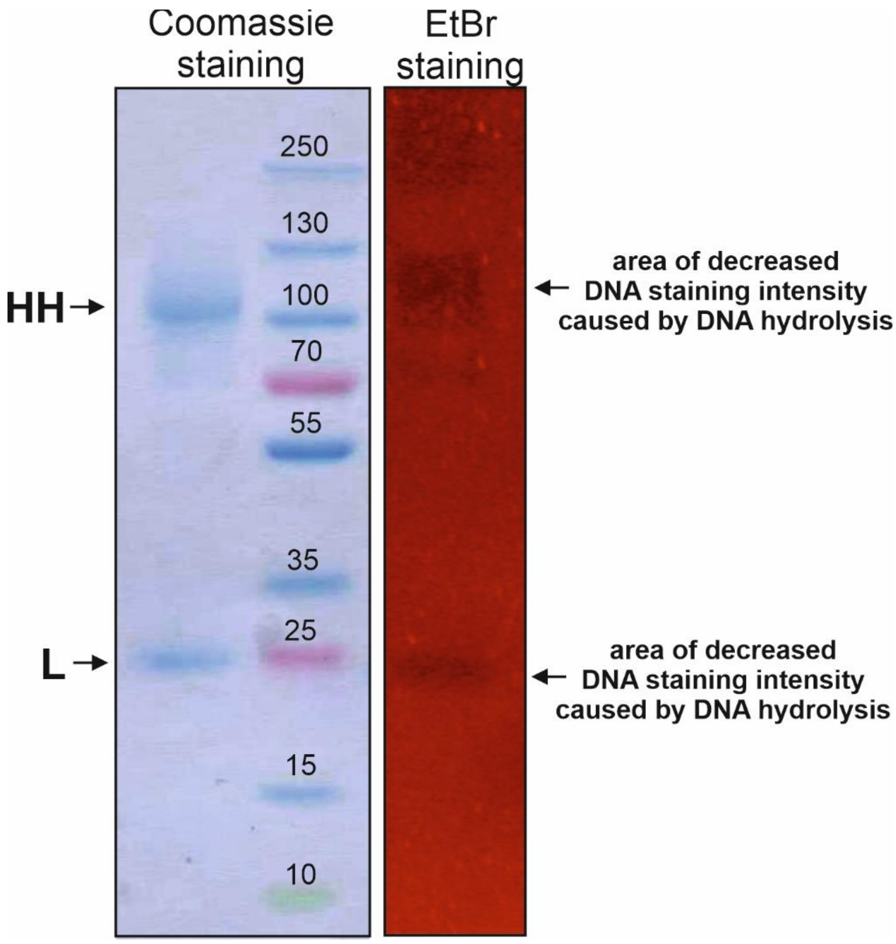

Figure S4. Zymographic (in-situ) analysis of DNA-hydrolyzing activity of IgG samples. IgG samples of SLE patients (20 μg protein/line) were separated in 4-18% gradient SDS-PAGE containing copolymerized calf thymus polymeric DNA. After washing from SDS and incubation for 48 h in buffer with optimal conditions for hydrolysis, the gel was stained with ethidium bromide (EtBr) (right panel). A decrease in DNA staining intensity occurred in areas with proteins with nuclease activity. The same gel was then stained with Coomassie G-250 (left panel) to visualize proteins. Disulfide bond-reducing agent (DTT, 0.1 M) was added to IgG samples but they were not boiled to prevent denaturation and maintain catalytic activity, so products of incomplete reduction of disulfide bonds are visible, in particular the dimer of two heavy chains (HH) (boiling destroys the HH dimer but leads to irreversible denaturation). As a result, it is shown that the position of areas of decreased DNA staining intensity caused by DNA hydrolysis corresponds to the position of the light chain and the HH dimer, indicating that both light and heavy chains exhibit DNase activity. In addition, this experiment confirms the absence of DNase impurities (they differ in molecular mass and, if they were present, other bands would be detected).

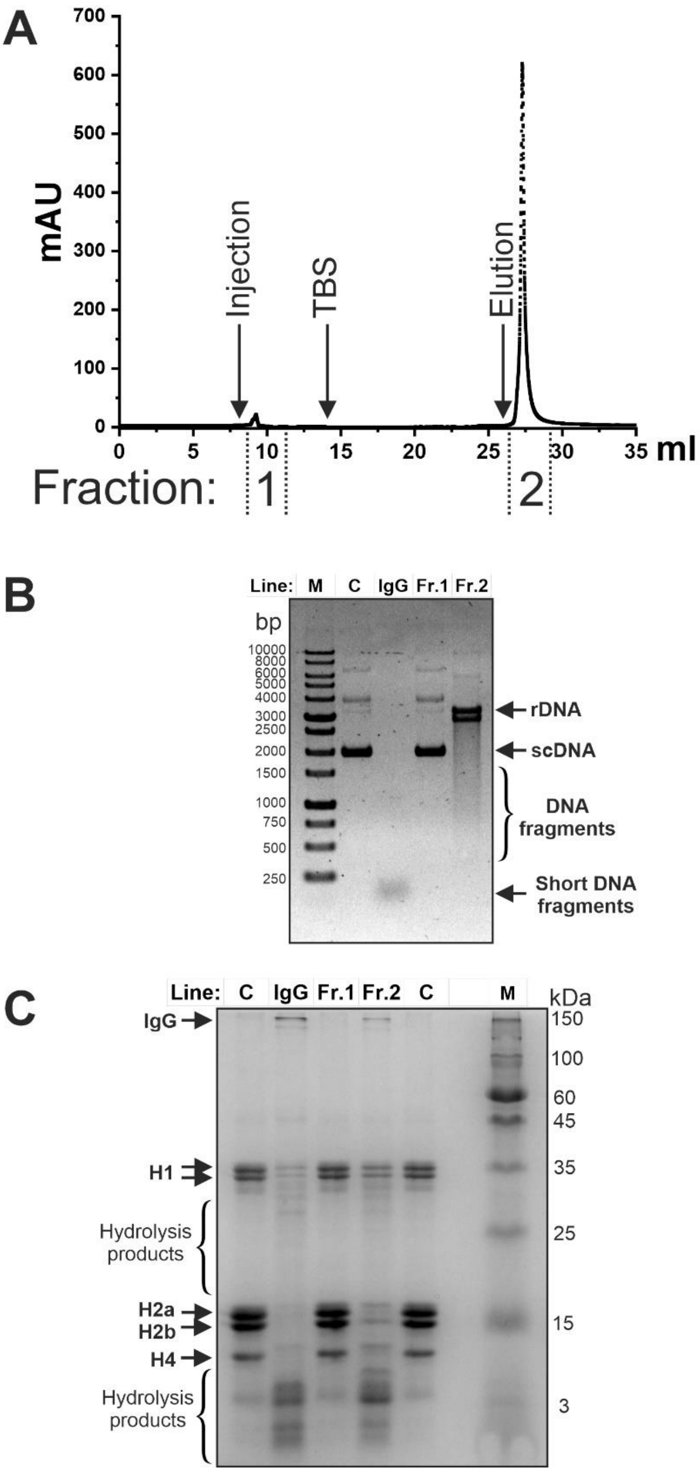

Figure S5. Analysis of catalytic activity of fractions after IgG sorption from the mixture. (**A**) An equimolar mixture of two IgG samples of SLE patients (IgG_{mix}) was applied to a rProtein G Seplife 4FF column. The flow-through fraction (Fraction 1) containing unbound proteins was collected. After washing the column with TBS, IgG was eluted with 100 mM Gly-HCl pH 2.6 (Fraction 2). (**B**) Analysis of plasmid DNA hydrolysis after 2 h of incubation with IgG_{mix} before chromatography and the obtained fractions by electrophoresis in 1% agarose gel. Plasmid DNA hydrolysis was evaluated by the transition of supercoiled (scDNA) to relaxed (rDNA) forms of plasmid DNA and shorter fragments. (**C**) Analysis of histone hydrolysis after 25 h incubation with IgG_{mix} before chromatography and the obtained fractions by 15% SDS–PAGE and Coomassie staining. (**B**,**C**) IgGmix actively hydrolyzed DNA and histones. Sorption of IgG from the mixture (Fraction 1) results in a loss of catalytic activity. IgG eluted from the column exhibits DNase or histone-hydrolyzing activity, but to a lesser extent due to partial denaturation under acidic conditions. In all cases: Line M indicates DNA or protein markers. Line C – control reactions without IgG. Line IgG – reaction with IgG_{mix} before chromatography. Lines Fr.1 and Fr.2 – reactions with Fractions 1 and 2, respectively.

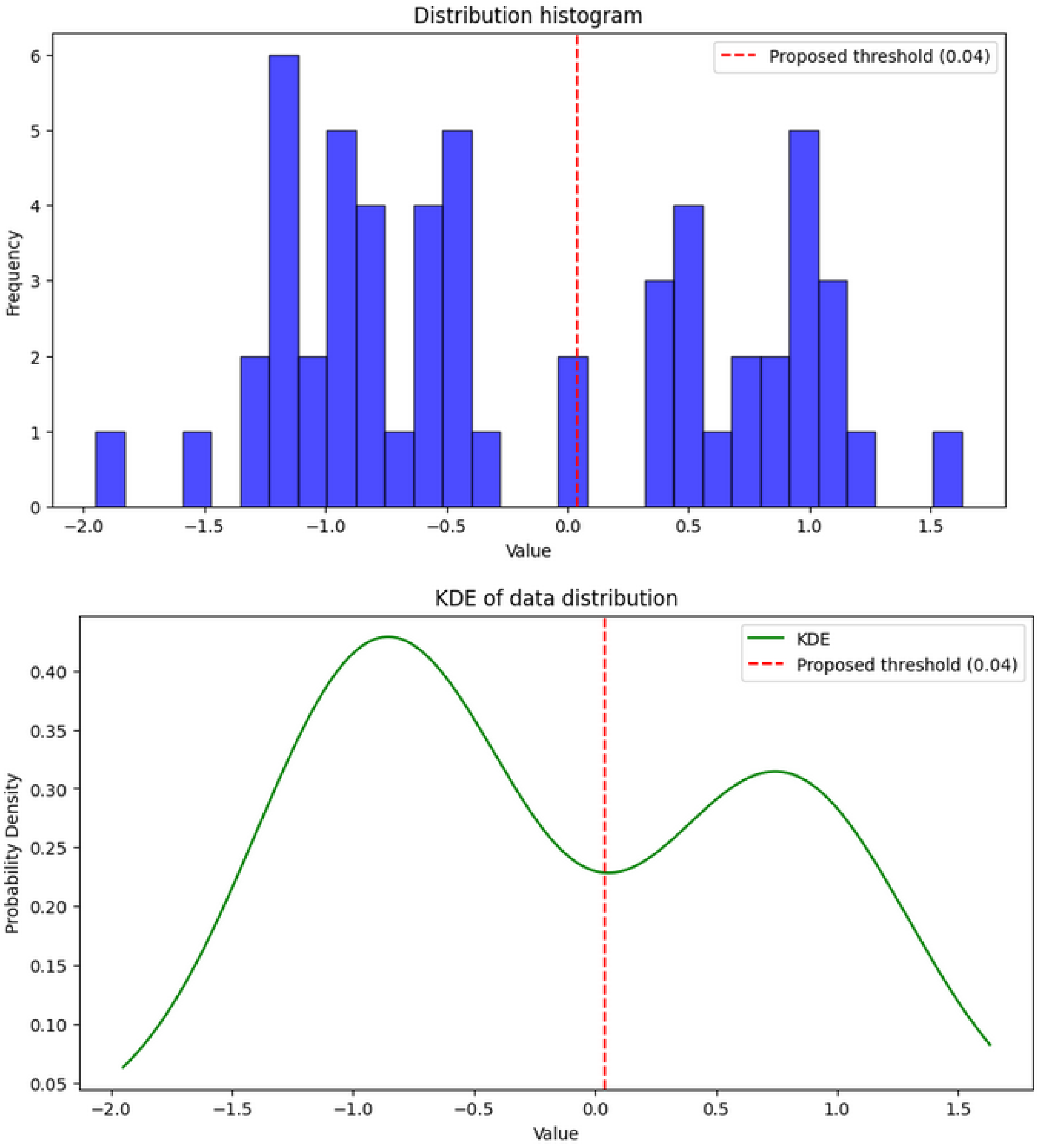

Figure S6. Analysis of the distribution histogram and smoothed distribution plot using the Kernel Density Estimation (KDE) algorithm of the level of IgG DNA-hydrolyzing activity in SLE.

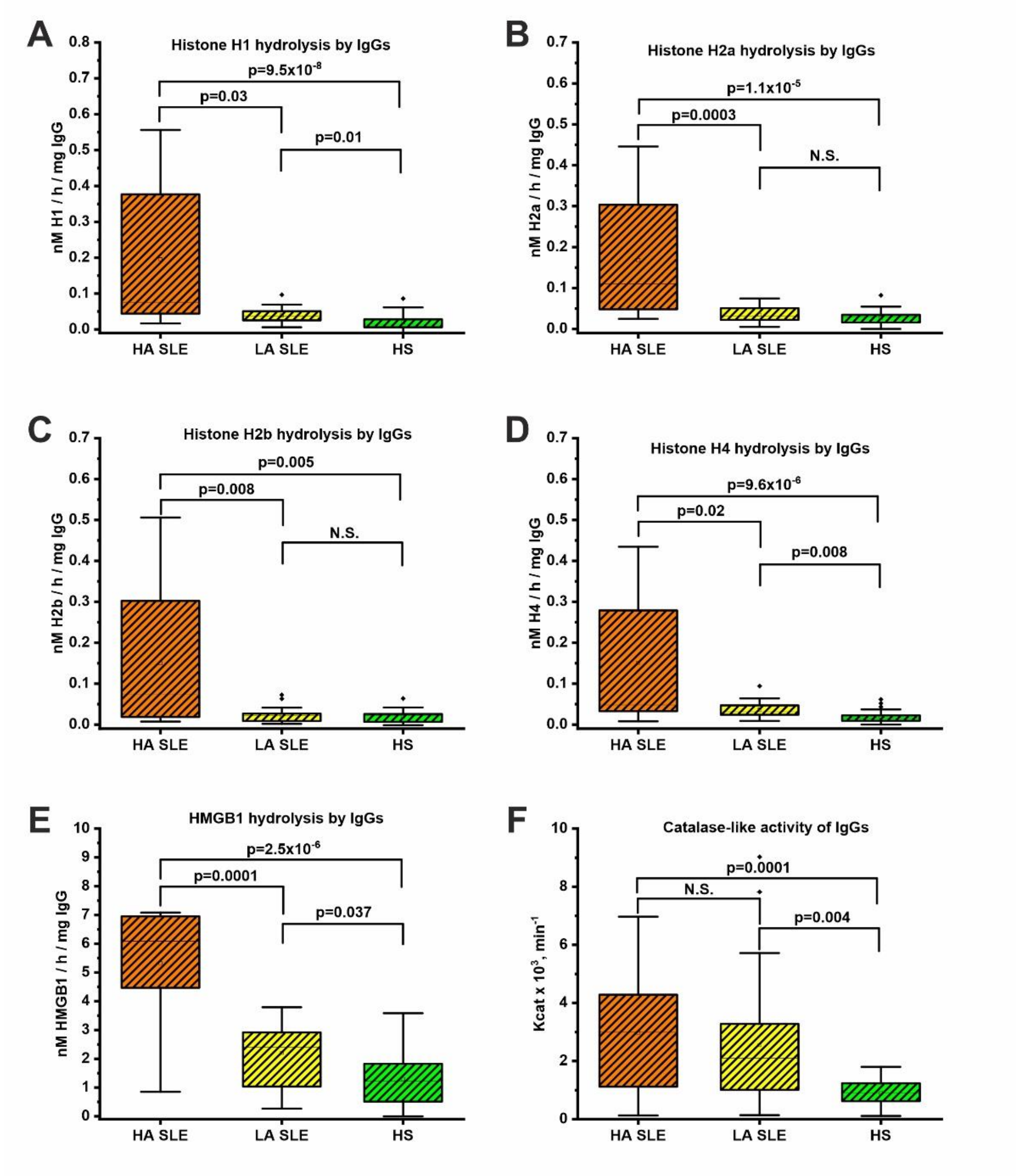

Figure S7. Comparison of proteolytic and catalase-like activities of IgG in HA SLE, LA SLE and HS groups. (**A-E**) Analysis of the histone-hydrolyzing activity of IgG in the hydrolysis of H1 (**A**), H2a (**B**), H2b (**C**), H4 (**D**) histones, and HMGB1 (**E**). (**F**) Analysis of the catalase-like activity of IgG. Statistical significance of differences was determined using the Kruskal-Wallis test.

Table S1. Raw data on the level of catalytic activity of antibodies in SLE patients and healthy individuals.

Sample #	Catalytic activity of IgG:							State
	DNA hydrolyzing	H1 hydrolyzing	H2A hydrolyzing	H2B hydrolyzing	H4 hydrolyzing	HMGB1 hydrolyzing	Catalase-like activity	
1	42.743	0.309	0.248	0.229	0.248	6.953	4.45	SLE
2	18.464	0.556	0.446	0.506	0.296	7.042	2.54	SLE
3	12.855	0.082	0.062	0.038	0.033	6.857	0.35	SLE
4	11.733	0.431	0.329	0.324	0.411	7.083	4	SLE
5	11.434	0.495	0.430	0.410	0.225	7.002	0.65	SLE
6	9.847	0.070	0.155	0.102	0.041	6.953	4.34	SLE
7	9.103	0.206	0.163	0.103	0.130	6.091	2.87	SLE
8	8.907	0.200	0.165	0.173	0.199	6.975	3.92	SLE
9	8.797	0.339	0.316	0.272	0.279	2.539	0.8	SLE
10	8.409	0.377	0.304	0.304	0.435	2.074	2.92	SLE
11	7.085	0.424	0.352	0.302	0.356	6.461	5.91	SLE
12	6.843	0.397	0.285	0.313	0.383	2.061	1.23	SLE
13	5.972	0.032	0.036	0.019	0.026	5.273	6.97	SLE
14	5.021	0.436	0.491	0.387	0.417	6.661	1.79	SLE
15	3.877	0.029	0.030	0.016	0.033	1.236	1.01	SLE
16	3.574	0.044	0.048	0.037	0.039	1.018	4.23	SLE
17	3.324	0.045	0.053	0.029	0.008		3.8	SLE
18	2.999	0.017	0.025	0.008	0.015	4.515	0.77	SLE
19	2.843	0.035	0.035	0.012	0.043	4.468	0.13	SLE
20	2.669	0.063	0.065	0.016	0.009	0.851	4.67	SLE
21	2.375	0.185	0.072	0.048	0.041	5.308	3.07	SLE
22	2.306	0.023	0.033	0.021	0.028	4.242	4.47	SLE
23	1.126	0.064	0.053	0.047	0.033		3.77	SLE
24	1.103	0.066	0.066	0.018	0.053	3.576	2.8	SLE
25	0.480	0.006	0.005	0.002	0.024		2.29	SLE
26	0.359	0.026	0.032	0.021	0.027		1.17	SLE
27		0.056	0.051	0.040	0.026			SLE
28	0.349	0.043	0.012	0.024	0.035			SLE
29	0.347	0.010	0.016	0.006	0.030	0.514	2.33	SLE
30	0.305	0.041	0.035	0.027	0.023	0.380	0.81	SLE
31	0.271	0.045	0.029	0.016	0.019	0.256	2.67	SLE
32	0.255	0.051	0.035	0.027	0.020		0.68	SLE
33	0.251	0.032	0.022	0.019	0.040		2.56	SLE
34	0.245	0.069	0.069	0.042	0.061		1.07	SLE
35	0.224	0.052	0.054	0.025	0.025		5.72	SLE
36	0.159	0.049	0.034	0.021	0.003		0.32	SLE
37	0.150	0.026	0.031	0.029	0.041		2.18	SLE
38	0.141	0.032	0.032	0.011	0.024		2.1	SLE
39	0.135	0.033	0.033	0.012	0.003	0		SLE
40	0.130	0.022	0.022	0.005	0.031	0.873	3.28	SLE
41	0.130						5.58	SLE
42	0.123						1.46	SLE
43	0.114	0.034	0.020	0.024	0.032	2.565	1.01	SLE

44	0.106	0.034	0.034	0.018	0.044	0	2.07	SLE
45	0.090	0.017	0.023	0.005	0.025	1.035	9.03	SLE
46	0.087	0.097	0.074	0.073	0.094	2.339	4.59	SLE
47	0.076	0.060	0.060	0.036	0.054	3.465	1.17	SLE
48	0.073	0.029	0.029	0.004	0.047	0	5.33	SLE
49	0.067	0.046	0.051	0.009	0.009		3.32	SLE
50	0.063					2.455	2.64	SLE
51	0.060					2.349	0.54	SLE
52	0.058	0.056	0.008	0.022	0.012		0.19	SLE
53	0.054					3.791	0.27	SLE
54	0.050	0.051	0.051	0.026	0.063	2.917	7.82	SLE
55	0.029	0.037	0.054	0.063	0.064	0.265	0.14	SLE
56	0.011	0.038	0.028	0.023	0.025		1.65	SLE
57	0.004	0.028	0.031	0.025	0.021		1.13	HS
58	0.012	0.021	0.050	0.030	0.044		1.36	HS
59	0.012					0.961	0.91	HS
60	0.005	0.015	0.019	0.018	0.023		0.13	HS
61	0.013	0.019	0.037	0.026	0.024			HS
62	0.023	0.000	0	0	0		0.67	HS
63	0.002		0.029	0.024	0.023		1.38	HS
64	0.524	0.086	0.082	0.064	0.062		0.47	HS
65	0.004	0.019	0.007	0.000	0.006	3.583	0.34	HS
66	0.001	0.061	0.054	0.042	0.052	0.340	0.79	HS
67	0.011	0.024			0.013		1.79	HS
68	0.002	0.002	0.023	0.026	0.005	2.163	1.58	HS
69	0.005	0.005	1E-03	0.006	0.009		0.72	HS
70	1.601	0.013	0.016	0.026	0.015		0.69	HS
71	5.054	0.034	0.032	0.022	0.030	2.981	1.30	HS
72	0.003	0.006	0.035	0.027	0.000		1.12	HS
73	0.011	0.002			0.012		1.18	HS
74	0.012	0.036	0.037	0.030	0.017	1.754	0.89	HS
75		0.035	0.032	0.026	0.014		1.80	HS
76	0.010	0.012	0.024	0.007	0.016	1.832	0.13	HS
77	0.012	0.010	0.022	0.004	0.022	1.086		HS
78	0.003					0	0.58	HS
79	1.669					2.065	1.14	HS
80	0.002	0.025	0.034	0.020	0.022	0.993	0.93	HS
81	0.908					0.377	1.01	HS
82	0.001					1.368	0.26	HS
83	0.160	0.009	0.008	0.008	0.019	0	0.11	HS
84	0.007		0.018	0	0	0.750		HS
85		0.024	0.037	0.023	0.037		1.47	HS
86	0.002					1.588	0.16	HS
87	0.014	0.000	0.008	0.003	0		0.79	HS
88	0.020	0.012	0.030	0.021	0.010			HS
89	0.033					1.564	0.81	HS
90	0.002	0.000				0	0.79	HS
91	0.034	0.009	0.020	0	0.011	0.647	1.47	HS

92	0.153	0.000			0	1.819	0.90	HS
93	0.002	0.000						HS

Note: Units of measurement for DNase, histone- and HMGB1-hydrolyzing activity of IgG: nM substrate / 1 h / 1 mg IgG. In case of catalase activity: kcat × 10^3, min^{-1}.
